# Hyponatremia causing factors and its association with disease severity and length of stay in COVID-19 patients: A retrospective study from tertiary care hospital

**DOI:** 10.1097/MD.0000000000035920

**Published:** 2023-11-10

**Authors:** Fazal ur Rehman, Syeda Tayyaba Rehan, Bakhtawar Jamal Rind, Komal Valliani, Muhammad Sohaib Asghar, Farrukh Omair

**Affiliations:** a Department of Medicine, Aga Khan University Hospital, Karachi, Pakistan; b Dow University of Health Sciences, Karachi, Pakistan; c Jinnah Sindh Medical University, Karachi, Pakistan; d School of Epidemiology and Public Health, University of Ottawa, Ottawa, Ontario, Canada; e Division of Nephrology and Hypertension, Mayo Clinic – Rochester, MN; f Consultant Nephrologist- Transplant, King Fahad Armed Forces Hospital, Jeddah, KSA.

**Keywords:** COVID-19, electrolyte imbalance, hyponatremia, length of stay, severity

## Abstract

The coronavirus disease-2019 (COVID-19) infection has taken the world by storm within a few months. Evidence has suggested that patients with electrolyte imbalances at baseline may have a longer duration of hospital stay. We aimed to determine the factors associated with hyponatremia on admission in COVID-19 patients and its impact on the length of stay. We conducted a retrospective study including 521 patients who tested positive for COVID-19 and had their electrolytes checked on admission from June 2020 to October 2020. Patients with sodium <135 mmol/l were included in the hyponatremic group and were compared against normonatremic patients. The severity of COVID-19 was found to be more prevalent in the case group as compared to control (38.3% vs 29.2%; 21.1% vs 17.7%). Hyponatremic patients stayed more than 5 days in hospital (56.3% vs 46.5%), and stayed longer in special care (23.4% vs 20.0%) as compared to controls. Hyponatremic patients as compared to control were more likely to have diabetes (47.9% vs 30.0%), hypertension (49.0% vs 38.5%), ischemic heart disease (20.7% vs 15.4%), chronic liver disease (2.7% vs 1.2%), and chronic kidney disease (9.6% vs 3.8%). Upon matching on the age, the adjusted odds of hyponatremia in COVID-19-positive patients were 1.9 times among diabetic patients. Moreover, COVID-19-positive patients suffering from CKD had a higher risk of developing hyponatremia (OR = 2.3, 95% CI: 1.1–5.6). The risk of hyponatremia among COVID-19-positive patients is statistically higher in patients with 1 comorbidity (OR = 1.9, 95%CI: 1.3–3.4). Hyponatremia on admission can be used to forecast the length of hospital stay and the severity of illness in COVID-19 patients.

## 1. Introduction

The severe acute respiratory syndrome coronavirus 2 infection has taken the world by storm within a few months. Due to rapid transmission, it has been declared as a pandemic in March 2020 by the World Health Organization and has affected over 767 million cases to date, with 6 million deaths worldwide.^[1]^ In Pakistan, over 1 million individuals have tested positive, and approximately 30,000 mortalities by the end of June 2023.^[2]^ The common manifestations of coronavirus disease-2019 (COVID-19) include fever, sore throat, dry cough, and body aches, and presentation with anosmia, and diarrhea has also been documented.^[3]^ The clinical spectrum of illness ranges from asymptomatic to mild respiratory illness to life-threatening respiratory failure, shock with multi-organ dysfunction, and death. The typical etiology of COVID-19 is favorable, however, some factors such as age above 65 years, male sex, underlying comorbidities, and preexisting lung conditions make the disease outcomes worse and increase the chances of mortality.^[4]^ Initial Chinese evidence has reported that electrolyte disorders may be present in the patient's initial report.^[3,5]^ Similar reports from other regions supported these findings.^[6,7]^ Some evidence has suggested that patients with electrolyte imbalances at baseline may have a longer duration of hospital stay.^[8]^ Such presentations have important implications not only for patient management but also for identifying potential pathophysiologic mechanisms underlying COVID-19, that could lead to novel therapeutic opportunities. Limited studies have collected the electrolyte data regarding the novel coronavirus-19, across the globe.^[9,10]^ Therefore, we aimed to determine the factors associated with hyponatremia on admission in COVID-19 patients and its impact on the length of stay.

## 2. Material and methods

### 2.1. Study design and setting

We conducted a retrospective, matched case-control study in the medicine department (primarily responsible for the management of COVID-19 patients) at the Aga Khan University Hospital (AKUH), which is a 563-bedded tertiary care center in Karachi, Pakistan.

### 2.2. Study participants

This retrospective study examined the records of 521 patients who tested positive for COVID-19, had their electrolytes checked on admission, and got admitted between June 2020 to October 2020 in the Department of Medicine, AKUH. Cases and controls were taken in a 1:1 ratio and were matched on age to reduce the effect of confounders.

### 2.3. Eligibility criteria

Inclusion Criteria: All adult patients with the diagnosis of COVID-19 by nasopharyngeal polymerase chain reaction (PCR) whose electrolyte levels were checked on hospital admission.

Exclusion criteria: COVID-19 cases with superimposing bacterial infections elicited by high procalcitonin levels, patients undergoing dialysis, patients who were already on diuretic therapy, and patients who were already managed in other facilities and transferred to the study centers for further management were excluded from this study.

### 2.4. Operational definitions

Mild Disease: COVID-19 without evidence of viral pneumonia or hypoxia.

Moderate Disease: COVID-19 patients with clinical signs of pneumonia (fever, cough, dyspnea, fast breathing) but no signs of severe pneumonia, including oxygen saturation (SpO2) > 90% on room air.

Severe Disease: Clinical signs of pneumonia (fever, cough, dyspnea, fast breathing) plus one of the following: respiratory rate > 30 breaths/min, severe respiratory distress; or SpO2 < 90% on room air.

Critical Disease: Symptoms onset within 1 week of a known clinical insult (i.e., pneumonia) or new or worsening respiratory symptoms. With Chest imaging: (radiograph, Computed Tomography scan, or lung ultrasound): bilateral opacities, not explained by volume overload, lobar or lung collapse, or nodules.

Hyponatremia: Serum sodium <135 mmol/L.

### 2.5. Data collection and study covariates

The study commenced after getting approval from the institute Ethical Review Committee. A waiver of informed consent was granted as this was a retrospective study, and all patients were discharged from the hospital. Patients admitted to the medicine department and fulfilling the inclusion criteria were enrolled. Data was collected on through a predesigned proforma questionnaire containing details of patient demographics characteristics (age, gender, comorbid conditions and Multimorbidity score) and clinical presentation characteristics (thromboembolic complication, pneumothorax complication, COVID-19 severity, coinfection, laboratory parameters) length of stay, type of hospital stay and clinical outcome, and laboratory parameters.

The outcomes of the study were serum sodium level and length of stay in COVID-19 positive patients. Cases were defined as patients who tested positive by sampling technique using nasopharyngeal swab run by PCR technique and had serum sodium (Na) <135 mmol/l (hyponatremia) at the time of admission. Controls were defined as patients who tested positive by sampling technique using nasopharyngeal swab run by PCR technique and had serum sodium level 135 mmol/L or above (normal-natremia) at the time of admission. Length of stay was recorded as a binary variable with the categories as hospital stay <5 days or >5 days

### 2.6. Statistical analysis

The analysis was carried out using the Statistical Package for Social Sciences, version 11.5. Initially, descriptive statistics and frequencies were generated. Matched sets were created in the datasets of each case and control. The univariate analysis was performed to identify differences between the cases and control group, using the t-test, and Pearson Chi-square test, as appropriate. Statistical significance for univariate analysis was demonstrated as a *P* value of <.25. Multivariable conditional logistic regression analysis was used to build a final model and adjusted matched odds ratios were calculated. Statistical significance was defined as a *P* value of < .05.

## 3. Results

### 3.1. Descriptive data

A total of 521 patient record files were reviewed for a duration of 5 months, from which 261 cases and 260 matched controls were identified as meeting the eligibility criteria and were included in the study. Table 1 shows the demographics and comorbidity status of the patients with hyponatremia and normal-natremia. More than half of the total study participants were above 55 years of age though, the proportion of cases among that category was greater than the controls (55.9% vs 48.8%). The proportion of males (75.1% vs 74.2%) was found similar in cases and controls. COVID-19 positive hyponatremia patients, compared with COVID-19 positive normo-natremia patients, were significantly more likely to have diabetes (47.9% vs 30.0%), hypertension (49.0% vs 38.5%), ischemic heart disease (IHD) (20.7% vs 15.4%), chronic liver disease (CLD) (2.7% vs 1.2%), and chronic kidney disease (CKD) (9.6% vs 3.8%). Nearly half of the controls had no comorbidity conversely, more cases had 1, 2, 3, or more comorbidities as compared to controls.

**Table 1:** Demographics and comorbidity status among Covid-19 patients according to their serum sodium level.

Variables	Serum Sodium level (mmol/L)		*P* value
	Cases (n = 261), n (%)	Controls (n = 260), n (%)	
Age			
<40 yr	40 (15.3%)	46 (17.7%)	.26
40–55 yr	75 (28.7%)	87 (33.5%)	
>55 yr	146 (55.9%)	127 (48.8%)	
Gender			
Female	65 (24.9%)	67 (25.8%)	.82
Male	196 (75.1%)	193 (74.2%)	
Diabetes			
No	136 (52.1%)	182 (70.0%)	<.01
Yes	125 (47.9%)	78 (30.0%)	
Hypertension			
No	133 (51.0%)	160 (61.5%)	.01
Yes	128 (49.0%)	100 (38.5%)	
Ischemic heart disease (IHD)			
No	207 (79.3%)	220 (84.6%)	.11
Yes	54 (20.7%)	40 (15.4%)	
Chronic obstructive pulmonary disease (COPD)			
No	259 (99.2%)	257 (98.8%)	.65
Yes	2 (0.8%)	3 (1.2%)	
Chronic liver disease (CLD)			
No	254 (97.3%)	257 (98.8%)	.21
Yes	7 (2.7%)	3 (1.2%)	
Chronic kidney disease (CKD)			
No	236 (90.4%)	250 (96.2%)	.01
Yes	25 (9.6%)	10 (3.8%)	
Multi-morbidity score			
None	85 (32.6%)	132 (50.8%)	.01
1 Comorbidity	71 (27.2%)	51 (19.6%)	
2 Comorbidities	56 (21.5%)	50 (19.2%)	
3 or more comorbidities	49 (18.8%)	27 (10.4%)	

Among clinical characteristics, more cases were found to have a complication score of 1 or 2 than in controls (11.1% vs 9.2%; 7.3% vs 4.2%) respectively (Table 2). For the severity of COVID-19, moderate or severe disease was found to be more prevalent in cases as compared to controls (38.3% vs 29.2%; 21.1% vs 17.7%). In contrast, mild or critical COVID-19 was more prevailing among controls than in the case group (38.5% vs 27.6%; 14.6% vs 13.0) respectively. A greater number of cases were found to have creatinine level > 1 mg/dL (55.9% vs 43.8%), stayed more than 5 days in hospital (56.3% vs 46.5%), and stayed longer in special care (23.4% vs 20.0%) as compared to controls.

**Table 2:** Clinical characteristics of study participants according to their serum sodium level.

Variables	Serum Sodium level (mmol/L)		*P* value
	Cases (n = 261) n (%)	Controls (n = 260) n (%)	
Thromboembolic complication			
No	238 (91.2%)	241 (92.7%)	0.52
Yes	23 (8.8%)	19 (7.3%)	
Pneumothorax complication			
No	255 (97.7%)	254 (97.7%)	0.99
Yes	6 (2.3%)	6 (2.3%)	
COVID-19 complication score			
None	213 (81.6%)	225 (86.5%)	0.23
1	29 (11.1%)	24 (9.2%%)	
2	19 (7.3%)	11 (4.2%)	
Severity of COVID			
Mild	72 (27.6%)	100 (38.5%)	0.03
Moderate	100 (38.3%)	76 (29.2%)	
Severe	55 (21.1%)	46 (17.7%)	
Critical	34 (13.0%)	38 (14.6%)	
Coinfections			
Undetermined	22 (8.4%)	35 (13.5%)	0.02
None	165 (63.2%)	167 (64.2%)	
Bacterial	48 (18.4%)	26 (10.0%)	
Fungal	8 (3.1%)	14 (5.4%)	
Viral	4 (1.5%)	1 (0.4%)	
Combination	14 (5.4%)	17 (6.5%)	
Creatinine at admission			
<1 mg/dL	115 (44.1%)	146 (56.2%)	0.01
>1 mg/dL	146 (55.9%)	114 (43.8%)	
Length of stay			
<5 d	114 (43.7%)	139 (53.5%)	0.02
More than 5 d	147 (56.3%)	121 (46.5%)	
Type of stay			
Ward	200 (76.6%)	208 (80.0%)	0.35
Special care	61 (23.4%)	52 (20.0%)	

COVID-19 = coronavirus disease-2019.

Regarding the length of stay, 52.9% of patients who stayed <5 days in the hospital, remained in the ward setting (Table 3). However, 67.3% of the patients who stayed more than 5 days in the hospital required special care. On the other hand, 56.4% and 50.4% of patients who took leave against medical advice (LAMA) and were discharged respectively; stayed below 5 days. While 65.8% of the patients who had their hospital stay more than 5 days expired.

**Table 3:** Type of stay and clinical outcomes according to the length of hospital stay.

Length of stay	Type of stay		Clinical outcome		
	Ward	Special care	Discharge	Expired	LAMA
<5 d	216	37	206	25	22
	52.9%	32.7%	50.4%	34.2%	56.4%
more than 5 d	192	76	203	48	17
	47.1%	67.3%	49.6%	65.8%	43.6%

### 3.2. Main results

In the multivariable model, diabetes, CKD, and multi-morbidity scores were found to be statistically significant (Table 4). Upon matching on the age, the adjusted odds of hyponatremia in COVID-19 positive patients are 1.9 times among diabetic patients. Moreover, COVID- 19 positive patients suffering from CKD had a higher risk of developing hyponatremia (OR = 2.3, 95% CI: 1.1–5.6). The risk of hyponatremia among COVID-19-positive patients is statistically higher in patients with 1 comorbidity (OR = 1.9, 95%CI: 1.3–3.4).

**Table 4:** Multivariable conditional logistic regression analysis of potential factors for hyponatremia among COVID patients.

Variables	Adjusted OR	95% CI
Diabetes		
No	Reference	
Yes	1.9	1.03–3.4
CKD		
No	Reference	
Yes	2.3	1.1–5.6
Multi-morbidity score		
None	Reference	
1 comorbid	1.9	1.3–3.4
2 comorbid	1.2	0.5–2.5
>=3 comorbid	1.4	0.5–3.5

## 4. Discussion

In this retrospective matched case-control study, we found that hyponatremic COVID-19 patients had a more severe illness, needed care in high-dependency units, and stayed longer in the hospital as compared to the COVID-19 patients who did not have any hyponatremia on admission. Our findings see eye to eye with other studies where hyponatremia was found to be positively correlated to the severity of COVID-19 disease, protracted length of hospital stays, and higher mortality.^[4,11,12]^ China A, et al and colleagues observed hyponatremia to be associated with severe disease and needed intensive care unit admission, ventilatory support, and longer hospital stay,^[13]^ while Sjostrom A, et al found hyponatremia on admission to be more strongly linked to the need of ventilatory support but not to death. However, he found hypernatremia to be more closely related to mortality in COVID-19 patients.^[14]^ Another study found hypernatremia to be less commonly associated with COVID-19 pneumonia and mortality as compared to hyponatremia.^[15]^

We found different comorbid conditions to be more prevalent in COVID-19 hyponatremic patients as compared to their counterparts; diabetes mellitus, hypertension, IHD, CKD, and CLD were the common medical comorbid conditions that were more frequently seen in cases as compared to the controls. In a study, Kang et al found ischemic heart diseases, diabetes mellitus, cerebrovascular diseases, and chronic kidney diseases to be more prevalent in hyponatremic patients.^[16]^ Mohan et al found 51% of Hypertensive, 16% of diabetic patients in his study population to be hyponatremic as compared to 27% of hyponatremic patients who didn’t have any comorbidities.^[17]^

The etiology of hyponatremia in COVID-19 patients is still being explored and is likely to be multifactorial with possibilities including decreased oral intake due to illness, presentation with diarrhea leading to electrolyte losses, syndrome of inappropriate antidiuretic hormone due to lung parenchymal injury as previously well-described in pneumonia and Adult Respiratory Distress Syndrome patients, impaired renal or cardiac function with disruption of renin-angiotensin-aldosterone system or use of medications to name a few.^[10,18]^ Interleukin-6, an inflammatory cytokine, released in response to the nonosmotic release of vasopressin causes hyponatremia and is positively related to disease severity.^[19]^

The association of hyponatremia with COVID-19 has been looked at in various parts of the world.^[4,15,20]^ However, data from the South Asian population is limited, where different genetic backgrounds may play a role. There are previously limited studies published from Pakistan which report conflicting results.^[21–23]^ A cross-sectional study by Sadiq A, et al with a sample size of 128 patients reported a significant association of hyponatremia with critical illness.^[21]^ However, our study found a stronger association with moderate to severe illness and a statistically insignificant difference in critical illness in the case and control groups. Saeed F, et al and colleagues studied the clinical characteristics and outcomes of dysnatremia but in contrast to our finding, they didn’t find any association of hyponatremia with the severity of COVID-19 pneumonia, though the length of stay was not observed as an outcome.^[22]^ The possible reason for their finding could be that it was a single-center observational study.

### 4.1. Limitations and recommendations

While our study reports different results from those conducted within the region, it is in alignment with international data. Our study had a robust sample size with matched controls for age. Although we established a link between hyponatremia and increased length of stay and severity in COVID-19, in-depth analysis of factors associated with hyponatremia including evaluation of etiology, volume status (hypovolemic, euvolemic, hypervolemic), duration prior to presentation in hospital (acute vs chronic), medication use (excluding diuretics), and other confounding variables were beyond the scope of this study. Larger, multicenter studies with diverse patient populations and evaluation of more extensive clinical and biochemical parameters are required to extrapolate more generalizable and uniform results. In addition, interventional studies are needed to address whether timely correction of sodium imbalances improves clinical outcomes in COVID-19.

## 5. Conclusions

Hyponatremia on admission can be used to forecast the length of hospital stay and the severity of illness in COVID-19 patients. Hyponatremia is found to be more prevalent in patients having underlying chronic renal failure or diabetes Mellitus. Early preventive measures can be taken to correct the serum sodium imbalance to avoid longer stay and complications in patients.

## Author contributions

**Conceptualization:** Fazal ur Rehman.

**Data curation:** Fazal ur Rehman.

**Formal analysis:** Muhammad Sohaib Asghar.

**Investigation:** Syeda Tayyaba Rehan.

**Methodology:** Bakhtawar Jamal Rind.

**Project administration:** Bakhtawar Jamal Rind, Farrukh Omair.

**Resources:** Farrukh Omair.

**Software:** Farrukh Omair.

**Supervision:** Komal Valliani.

**Validation:** Komal Valliani.

**Visualization:** Komal Valliani.

**Writing – original draft:** Syeda Tayyaba Rehan.

**Writing – review & editing:** Muhammad Sohaib Asghar.
